# Anthropophagy and Ecological Bridges: Blood-Meal Patterns of Invasive *Aedes albopictus* (Skuse, 1894) and Native *Aedes aegypti* Linnaeus, 1762 and Their Implications for Arbovirus Emergence in Central Africa

**DOI:** 10.3390/tropicalmed11060143

**Published:** 2026-05-25

**Authors:** Armel N. Tedjou, Christophe R. Keumeni, Aurélie P. Yougang, Flobert Njiokou, Jo Lines, Sian E. Clarke, Charles S. Wondji, Basile Kamgang

**Affiliations:** 1 Department of Medical Entomology, Centre for Research in Infectious Diseases, Yaoundé P.O. Box 15391, Cameroonaurelie.yougang@crid-cam.net (A.P.Y.);; 2 Department of Animal Biology and Physiology, Faculty of Science, University of Yaoundé I, Yaoundé P.O. Box 337, Cameroon; 3 Disease Control Department, Faculty of Infectious Tropical Diseases, London School of Hygiene and Tropical Medicine, London WC1E 7HT, UK; 4 Vector Biology Department, Liverpool School of Tropical Medicine, Pembroke Place, Liverpool L3 5QA, UK

**Keywords:** *Aedes albopictus*, *Aedes aegypti*, blood meal, monkeys, bats, arboviruses, Cameroon

## Abstract

*Aedes (Ae.) aegypti* and *Ae. albopictus* are important vectors of arboviruses. Yet their blood-feeding pattern remains poorly characterised in Africa, including Cameroon. In this study, we characterised the blood-meal sources in both species collected from vegetation, household surroundings, and animal cages across four urban sites, one rural site, and a zoo-botanical garden where humans and animals in captivity are the main hosts. Overall, *Aedes* mosquitoes represented about half of 10,054 female mosquitoes collected, with *Ae. albopictus* strongly dominating *Ae. aegypti* among 5001 *Aedes* females, and only 5.95% of females visibly blood-fed. Sequencing a 748 base pairs (bp) fragment of the cytochrome oxidase I gene from 156 blood-fed abdomens yielded 126 high-confidence host assignments, of which 98.25% were humans, indicating a strong anthropophagic pattern in both species. Unpredictably, two *Ae. albopictus* individuals had fed on a baboon (*Papio anubis*) and a frugivorous bat (Pteropodidae)*,* as confirmed by bio informatic analyses, highlighting the species’ opportunistic blood-feeding nature and providing preliminary molecular evidence consistent with a potential bridge-vector role in this setting. Despite the extreme anthropophagy of both species observed, results indicate that *Ae. albopictus* could also serve as a bridge vector enabling spillover of enzootic viruses to humans, including urbanised settings where wild animals are present. These findings emphasise the urgent need for enhanced arbovirus surveillance in Central Africa using a One Health approach.

## 1. Introduction

Dengue, chikungunya, yellow fever, and Zika are emergent or reemergent arboviral diseases that count among the major global health threats in the world [1]. This could be mainly attributed to the rapid colonisation of *Aedes (Ae.) aegypti* and *Ae. albopictus* into new ecological niches, particularly shaped by major environmental factors like climate change and the rapid urbanisation growth of cities [2,3]. Among these diseases, dengue is the most widespread and significant arboviral-borne disease globally, with roughly half the world’s population at risk and up to 400 million cases detected annually [4]. In 2024, dengue outbreaks hit record highs, with around 14 million cases and 9000 deaths, most notably in Latin America and East Asia [5]. Countries in Africa, such as Nigeria, Cameroon, and Burkina Faso, also experienced outbreaks, although the true burden may be underestimated because overlapping symptoms with malaria lead to misdiagnosis and underreporting [6,7].

The invasion of *Ae. albopictus* into Africa, Europe, and the Americas has reconfigured transmission risks by introducing a vector characterised by both urban anthropophagy and opportunistic feeding behaviours. The ability of this species to modify its feeding ecology is directly correlated to its epidemiological relevance [2,8,9].

Blood-meal analysis can provide distinctive insight into the understanding of vector–host interactions by using advancements in DNA barcoding of mitochondrial cytochrome oxidase I (COI) and cytochrome b (Cyt. b) genes to identify vertebrate hosts from wild-caught blood-fed female mosquitoes [10,11]. High-throughput sequencing now makes it easier to identify mixed or partially degraded meals, revealing underappreciated levels of multiple host feedings [9]. This tool can improve the blood-meal index and give quantitative foraging ratios that include host availability, enabling stronger epidemiological modelling. Previous studies have underscored that feeding behaviours are context-dependent, influenced by host density, landscape, and the historical population dynamics of mosquitoes [9,12].

Existing data on the pattern of *Ae. albopictus’s* feeding behaviour contrasts between the strictly anthropophilic and plastic generalist nature of the mosquito. In dengue-endemic regions of Asia, human blood meals can exceed 90% [13,14]. However, in North America and Europe, less than 20% of meals may derive from humans, with pets and wildlife contributing substantially to the species’ dietary plasticity [15]. In Africa, host-feeding rates on different hosts vary depending on the ecological contexts and collection methods [12,16]. This variation complicates epidemiological risk predictions and highlights the necessity for further research on the origin of blood meals ingested by mosquitoes, specific to each ecological setting.

Occasional feeding of mosquitoes on non-human primates or other vertebrates has contributed to the assumption that *Ae. albopictus* could act as a bridge vector, allowing spillover of sylvatic cycles of dengue fever virus or other zoonotic arboviruses to spread to humans [14]. Recent studies highlight the unpredictability of such rare biological events in driving major outbreaks [9].

The emergence and proliferation of *Ae. albopictus* in Africa since 1990 [17] coincides with rapid and unplanned urbanisation in many African cities [15,18,19,20]. In Cameroon, significantly high anthropophagy (>95%) in *Ae. albopictus* has already been documented [12], adding to the established role of *Ae. aegypti* as an important dengue vector in Africa. While predominantly human-biting, *Ae. albopictus* occasionally feeds on wildlife, creating potential ecological bridges for zoonotic arboviruses [21,22,23]. However, systematic blood-meal monitoring remains absent from vector surveillance programmes in Africa [8].

Taken together, the literature underscores the urgent need to refine our understanding of *Aedes* host feeding in Africa, where rapid urbanisation and environmental change converge with changing *Aedes* species distributions, examining patterns of anthropophagy as well as rarer wildlife or opportunistic feedings. In the present study, we characterised the blood-feeding patterns of *Ae. aegypti* and *Ae. albopictus* in Cameroon using molecular and sequencing assays on the host of DNA to quantify anthropophagy levels, detect evidence of alternative wildlife hosts, and assess the potential role of invasive *Ae. albopictus* as an ecological bridge for arboviral diseases emergence or re-emergence at the human–wildlife interface.

## 2. Materials and Methods

### 2.1. Study Sites

**Figure 1:**
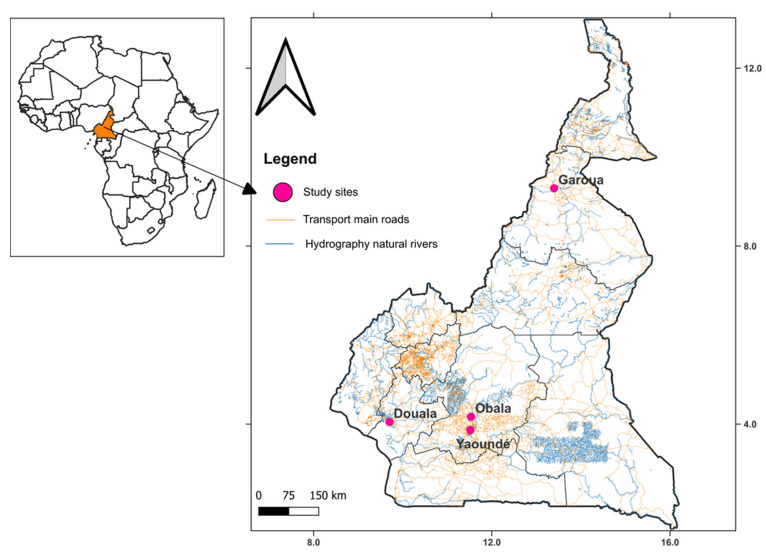
Map of Cameroon showing collection sites.

Adult mosquitoes were collected in cities in Cameroon (Figure 1), including Garoua, Yaoundé, Douala, and Obala, in different years (2019 to 2020 and 2023 to 2024) during different seasons (rainy and dry), different times of the day (early in the morning, 06 to 11 a.m., and in the afternoon or early evening, 03 to 07 p.m.), and mainly in urban environments (Table 1). In all study sites, adult mosquito collections were performed exclusively in outdoor environments, targeting resting sites in vegetation, near used tyres, tree holes, and animal enclosures. No indoor or intradomiciliary sampling was conducted during this study based on previous observations over the past two decades, which have reported *Aedes (Ae.)* vectors as outdoor-preferred mosquitoes [18,24,25,26,27].

Furthermore, this study was not designed as a standardised, cross-sectional surveillance survey. Rather, it is a descriptive, opportunistic study that capitalises on blood-fed *Aedes* females collected across multiple independent entomological surveys conducted between 2019 and 2024. The zoo-botanical garden dataset (2019–2020) was collected specifically to investigate blood-meal diversity in a wildlife–human interface setting, while the urban and rural site data (2023–2024) were obtained from routine entomological monitoring activities. Differences in seasonal timing, collection year, and site-level ecology across the dataset therefore reflect the real-world heterogeneity of mosquito habitats in Cameroon rather than a systematic experimental design. All comparative analyses between sites and species are consequently descriptive in nature, and site-to-site comparisons should be interpreted with caution, given the non-standardised sampling framework.

This study started in 2019 in the zoo-botanical garden of Mvog-Betsi (ZGMB) in the peri-urban zone of Yaoundé city, and was further expanded to other cities due to the availability of mosquito samples collected later in 2023–2024 in other cities, which could explain the differences observed in the seasons, day period, and environments of collections. The zoo-botanical garden of Mvog-Betsi in the peri-urban zone of Yaoundé city, owing to the presence of various non-human primates and other vertebrates that could serve as diverse blood meal hosts. The zoo-botanical garden of Mvog-Betsi (3°51′ N, 11°29′ E) is a protected wildlife area of 2.0647 ha where animals are quarantined and prepared either for export or for reintroduction into the wild. Three classes of animals housed in enclosures include several species belonging to the class of mammals (lion, bush pig, blue duiker, monkey), birds (guinea fowl, African grey parrot, boat eagle, macaco), and reptiles (Nile crocodile, dwarf crocodile, furrowed turtle, Gabon viper, African python, cobra). Amongst the mammals, non-human primates are the most represented, with more than 77 individuals. At the same time, the zoo-botanical garden has diverse flora with nearly 20 families and 24 species spread over its space, including some fruit species that are used to feed captive animals. Previous studies have documented several fruit trees that attract or serve as food for bats, including grapefruit, guava, soursop, and avocado plants, which are found in the garden [28,29]. The presence of vegetation, coupled with natural (tree hollows) and artificial (watering holes) breeding sites, provides favourable development and resting places for mosquitoes. With approximately 30 employees, the garden receives an average of 64,000 visitors per year [28], which, together with the presence of vertebrates, represents a wide variety of hosts for blood meals. In the zoo-botanical garden, mosquitoes were collected in the main area of the park, mainly around the cages of animals, the trees, and close to the vegetation. In other cities, mosquitoes were collected alongside main roads, mainly in used tyres, vegetation, trees, and rock holes. Urban environments were characterised by densely crowded populations and unplanned urbanisation, with dwellings less surrounded by vegetation. In contrast, rural environments featured houses more extensively surrounded by vegetation, an abundance of natural breeding sites like tree holes, and a population more sparsely and widely dispersed in the area.

### 2.2. Mosquito Collection and Identification

Adult mosquitoes were sampled using the modified CDC Back-pack Aspirator Model 1412 (John Hock Company, Gainesville, FL, USA) [30], the Improved Prokopack Aspirator Model 1419 (John Hock Company, Gainesville, FL, USA), and the BioGents-Sentinel trap (Biogents AG, Regensburg, Germany), following the manufacturer’s instructions for each mosquito trap. Collected adult mosquitoes were brought back to the insectary, knocked down at −20 °C, identified using morphological identification keys [31,32] under a binocular microscope (Leica Microsystems, Wetzlar, Germany), and sorted and conserved per genus. Across all sites, the collected *Aedes* females were morphologically identified as either *Ae. albopictus* or *Ae. aegypti*; no other *Aedes* species were collected. Only *Aedes* females were grouped per species (*Ae. albopictus* and *Ae. aegypti*) and repletion status (blood-fed and unfed). Blood-fed females were stored individually in a 1.5 mL Eppendorf tube containing silica gel (desiccant) and kept at −20 °C for molecular analysis.

### 2.3. DNA Extraction, Amplification, and Sequencing of the Cytochrome Oxidase Genes for Blood Meal Identification

The abdomen of each *Aedes* spp. female mosquito, containing blood residue, was separated from the body with tweezers and placed into a 1.5 mL Eppendorf tube, and homogenised in 200 µL of grinding buffer (comprising 180 µL of ALT buffer and 20 µL of proteinase K) using a pestle with an automatic grinder. Total DNA was extracted from blood-fed abdomens of females of *Ae. aegypti* and *Ae. albopictus* species using Qiagen DNAeasy Blood and Tissue kit (QIAGEN Ltd., Manchester, UK), following the manufacturer’s instructions, and used as a template for blood meal identification through the amplification of cytochrome C oxidase I (COI). More specifically, published vertebrate primers targeting the vertebrate-specific cytochrome C oxidase I (COI), the mammalian-specific cytochrome b (Cyt-b) [33], and the human-specific cytochrome C oxidase (COXI) [34], were used to amplify the portion of the cytochrome oxidase gene, using a polymerase chain reaction (PCR) targeting the amplification of a fragment of 228 base pairs (bp), 748 bp, and 772 bp, for humans, vertebrates, and mammals, respectively, with each corresponding primer pair (Table 2).

Cytochrome oxidase gene was amplified in a 15 µL reaction mixture comprising 1X of KAPA A buffer, 25 mM of MgCl_2,_ 10 mM of dNTPs, 10 µM of each primer, 5U/µL of KAPA Taq polymerase, and 1 µL of DNA template of each blood-fed female. The reaction mixture undergoes thermal amplification with an initial denaturation phase at 95 °C for 5 min, followed by 35 cycles of denaturation at 94 °C for 40 s, hybridisation at 54 °C for 40 s, and an elongation at 72 °C for 1 min, and a final elongation step at 72 °C for 10 min. PCR products of the COI gene were subsequently purified following the ExoSAP-IT (Thermo Fisher Scientific Inc-Scheepsbouwersweg 1b—Postbus 4—1120 AA Landsmeer) manufacturer’s recommendations. The purified amplicons were finally sequenced commercially via the Sanger method.

### 2.4. Phylogenetic and Statistical Analysis

Raw sequences were manually inspected, edited when necessary, and aligned using Clustal in BioEdit software version 5.0.9. High-quality COI sequences were then formatted in FASTA and batch-queried against the National Center for Biotechnology Information (NCBI) nucleotide database (https://www.ncbi.nlm.nih.gov/genbank/ (accessed on 06 January 2026)) and BOLD SYSTEMS version 2.5 (http://www.barcodinglife.org), using the BLASTN program (NCBI BLAST+ v2.15.0). Batch mode enabled simultaneous analysis of all sequences. The following key output metrics were recorded for each sequence: the query coverage (QC, the percentage of the query sequence included in the alignment), the percentage of identity (PI, the proportion of identical nucleotides across the alignment), the E-value (the expected number of alignments with a similar score that could occur by chance—values << 1 indicate highly significant matches), the bit score (the normalised alignment score—higher scores denote better alignment), and the accession number (the unique identifier of the database sequence producing the alignment). For downstream host assignment, hits were filtered with the following stringent criteria: (i) query coverage ≥ 90%, (ii) percent identity ≥ 90%, (iii) E-value ≤ 1 × 10^−20^. Species-level identification was assigned when the top hit corresponded unambiguously to a single vertebrate species under these cut-offs. Otherwise, the blood meal was classified at the lowest common taxonomic level shared by the top five hits [35].

A phylogenetic tree was analysed by the evolutionary analysis by the maximum likelihood method. Briefly, the bootstrap consensus tree from 1000 replicates was taken to represent the evolutionary history of the taxa analysed, where branches corresponding to partitions reproduced in less than 50% of replicate trees are collapsed. The percentage of replicate trees in which the associated taxa clustered together (1000 replicates) is shown [36]. The initial tree for the heuristic search was selected by choosing the tree with the superior log-likelihood between a Neighbour-Joining tree and a Maximum Parsimony tree [37]. The Neighbour-Joining tree was generated using pairwise distances computed using the Tamura-Nei model [38]. Evolutionary analyses were conducted in MEGA12, utilising up to 4 parallel computing threads [39].

The blood meal index for each host species was calculated for each mosquito species. The proportion of engorged mosquitoes that contain a blood meal from a specific host is indicated. An index above 50% is classified as high, whereas an index below 50% is classified as low, as previously described by [33].

## 3. Results

### 3.1. Mosquito Abundance

**Figure 2:**
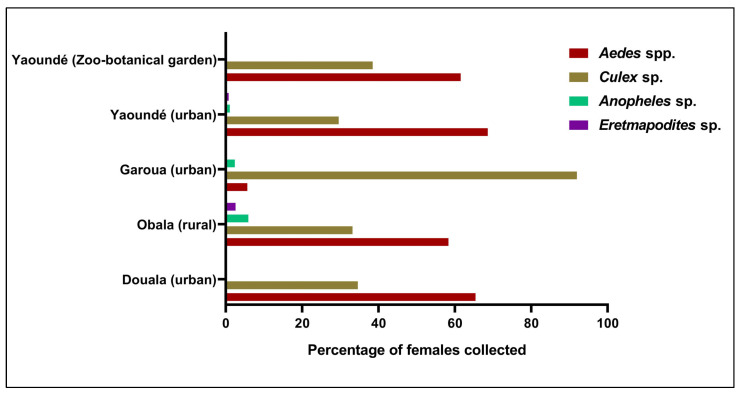
Relative abundance of adult female mosquitoes by genus across sampling sites in Cameroon (2019–2024). Horizontal bars represent the percentage contribution of each mosquito genus to the total number of females collected per site. Collections were conducted during 2019–2020 at the Yaoundé Zoo-botanical garden and during 2023–2024 at all other sites.

A total of 10 054 female mosquitoes were collected across all sites, from which *Aedes* species were the most abundant, representing nearly half (49.74%). In addition to *Aedes* spp., other mosquito genera collected included *Culex* (48.72%), *Anopheles* (1.21%), and *Eretmapodites* (0.33%) (Figure 2; Supplementary Table S1).

A total of 5001 female *Aedes* mosquitoes were collected across the five locations, comprising 83.4% (*n* = 4171) *Aedes albopictus* and 16.6% (*n* = 830) *Aedes aegypti* (Table 3).

In locations where both species were found together, *Ae. albopictus* was significantly more abundant than *Ae. aegypti*, except in Douala in the Littoral Cameroon. In Garoua, in the Northern part of the country, no *Ae. albopictus* were found during our collections.

### 3.2. Abundance of Blood-Fed Aedes spp. Females

Overall, 298 blood-fed females were recorded, representing 5.95% of the total number of *Aedes* collected. In addition to the higher overall abundance of *Ae. albopictus*, a higher proportion of blood-fed females was also recorded for this species (6.32%; 264/4171) compared to *Ae. aegypti* (4.09%; 34/830) (ꭓ^2^ = 5.77, *p* = 0.016); however, this varied markedly between sites. The highest percentage of blood-fed females was recorded in one of the most densely populated urban areas of Yaoundé for *Ae. aegypti* (9.89%; 18/182), and in the zoo-botanical garden of Mvog-Betsi for *Ae. albopictus* (15.62%; 150/960) (Table 3). Notably, no *Ae. aegypti* were collected in the zoo-botanical garden of Mvog-Betsi.

### 3.3. Identification of Blood-Meal Hosts

**Figure 3:**
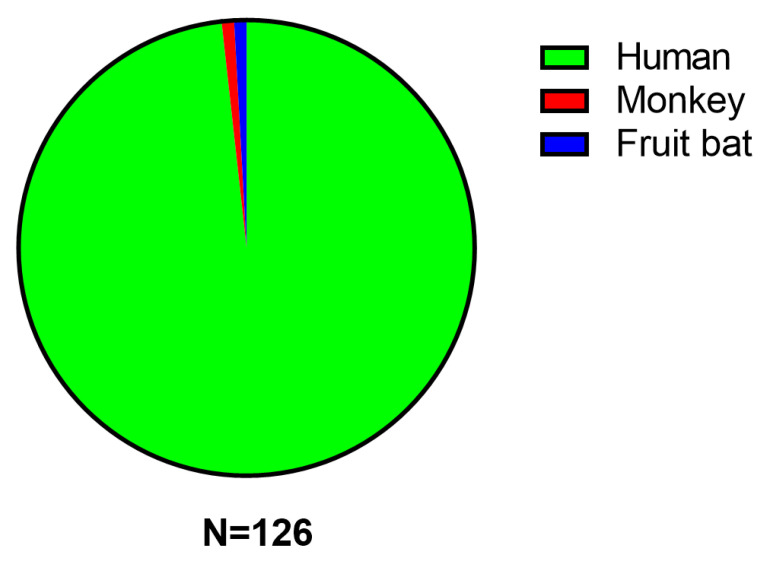
Source of vertebrate blood meal sources identified from Aedes mosquitoes in Cameroon. Pie chart showing the overall proportion of different vertebrate hosts identified from engorged *Aedes* females using mitochondrial COI barcoding. Most blood meals were derived from humans (green, >98%), with rare meals from a monkey (*Papio anubis*, red) and a fruit bat (Pteropodidae, blue). These findings highlight the predominantly human-feeding behaviour of *Aedes* mosquitoes in Cameroon, while underscoring sporadic exploitation of wildlife that may facilitate zoonotic arbovirus spillover.

**Figure 4:**
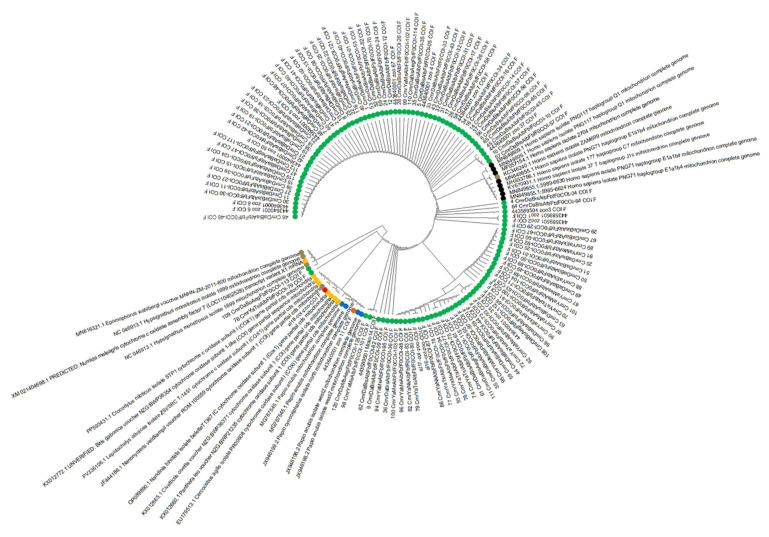
Evolutionary analysis by the Maximum Likelihood method. The phylogeny was inferred using the Maximum Likelihood method and the Tamura-Nei (1993) model of nucleotide substitutions, and the tree with the highest log likelihood (−3029.04) is shown. The percentage of replicate trees in which the associated taxa clustered together (1000 replicates) is shown next to the branches. The NJ tree was generated using a matrix of pairwise distances computed using the Tamura-Nei (1993) model. The tree is drawn to scale, with branch lengths measured in the number of substitutions per site. There was a total of 615 positions in the final dataset. Branch colors indicate sequence groups: green, local isolates; black, human reference strains; blue, monkey reference strains; brown, bat reference strains; yellow, outgroup sequences.

Blood meal sources were determined in both species by amplifying and sequencing the mitochondrial cytochrome c oxidase subunit I (COI) gene from blood-fed abdomens (*n* = 156). COI barcode sequences obtained yielded 126 confident assignments by BLAST against the NCBI nucleotide database. The rest of the sequences were unusable for analysis, due to several factors, likely poor amplicon purification or sequencing failure. Matches were overwhelmingly identified to human (*Homo sapiens* Linneaus, 1758) mitochondrial sequences (124/126; 98.25%) (Figure 3), with a query coverage (QC) of 100%, percentage of identity (PI) varying from 90 to 99.9% (Supplementary Table S2), and E-values close to zero, consistent with host identification. This result suggests strong anthropophagy for both *Ae. albopictus* and *Ae. aegypti* populations in urban environments in the prospective locations. Critically, among successful blood meal sequences, two yielded COI barcodes, consisting of one corresponding to fruit bats within the Pteropodidae family (*Hypsignathus monstrosus* Allen, 1861, *Epomophorus wahlbergi,* Sundevall, 1846) (QC = 93%, E-value = 0, PI = 90.97%), and the other corresponding to monkey sequences within the Cercopithecidae family (e.g., *Papio anubis* Lesson, 1827, and *Papio cynopcephalus* Linnaeus, 1758), with high query coverage (QC = 100%), PI more than 98%, and E-value close to zero compared with reference GenBank entries (Supplementary Table S2). Maximum-likelihood phylogenetic analysis of the same COI region summarised these BLAST identifications, by recovering three host-derived clades (human, bat, and monkey). Blood-meal sequences assigned to humans *Homo sapiens* clustered tightly with human COI references (green clade in Figure 4), whereas the two non-human meals grouped with Pteropodidae (bats) barcodes (orange clade in Figure 4) and Cercopithecidae (Old World monkey) references (blue clade in Figure 4), corroborating the BLAST calls.

Node support values across these major clades exceeded standard thresholds, indicating strong phylogenetic resolution. Importantly, no mosquito-derived sequences grouped spuriously with distantly related references, reinforcing the reliability of these host assignments. Thus, both similarity-based and tree-based approaches converge on a strongly anthropophagic feeding pattern, punctuated by rare opportunistic feeds on nearby wildlife, particularly of *Ae. albopictus* populations.

## 4. Discussion

In the present work, we evaluated the blood-feeding pattern of *Ae. albopictus* and *Ae. aegypti* in densely populated urban and rural environments, as well as in a protected area for wildlife. Our results provide strong evidence that *Aedes* spp. mosquitoes in urban settings of Cameroon overwhelmingly feed on humans, a pattern corroborated by both sequence similarity analysis and phylogenetic placement of blood-meal COI barcodes. More than 98% of identified blood meals from both species, *Ae. albopictus* and *Ae. aegypti*, matched to humans (*Homo sapiens*) in all the sites, which is consistent with observations previously made in Cameroon [12] and elsewhere in Africa, including Senegal [16], Kenya [40], as well as other parts of the world, including Italy [41], La Reunion [42], Thailand [43], and the USA [44]. However, blood meals derived from a frugivorous bat (Pteropodidae) and an Old-World monkey (Cercopithecidae) in *Ae. albopictus* collected in our study, although rare, confirms the opportunistic feeding characteristic of this mosquito species [45].

The numerical dominance of *Ae. albopictus* in our collections (83.4% overall) could mirror the method of collection used, whereby *Aedes* species were collected in vegetation resting areas, which has previously been shown to be significantly associated more with the presence of *Ae. albopictus* than *Ae. aegypti* [19,46,47]. Our data also showed that the prevalence of blood-fed females varied substantially by location, ranging from zero in Garoua and Obala to over 15% in the zoological park. Environmental factors such as vegetation cover, human density, and resting site availability could likely drive this heterogeneity [27,46], echoing previous data from the Andaman and Nicobar islands’ heterogeneous landscapes, where *Ae. albopictus* maintained high human-feeding rates in forest-fringe sites, but fed even more frequently on humans in densely populated urban areas [13]. Such patterns indicate that *Ae. albopictus* populations could modify their feeding behaviour according to local ecological conditions, thereby potentially increasing the risk of arbovirus transmission in regions where humans and wildlife live in sympatry. In Yaoundé, higher blood feeding in *Ae. aegypti* compared with *Ae. albopictus* (the reversal of the pattern seen elsewhere) could be attributed to collections mainly made in densely populated neighbourhoods, where *Ae. aegypti* tends to remain the predominant species, despite the invasiveness of *Ae. albopictus* [27,46]. Such micro-ecological variations, also documented in Korea [48], may have direct implications for the design of vector surveillance within cities where monitoring should not assume species-level distribution, but account for fine-scale neighbourhood-level differences in species abundance and feeding behaviour. Recognising this local heterogeneity is essential for designing targeted vector-control strategies that focus on high-risk areas where human–mosquito contact is most likely to facilitate disease transmission.

Non-human blood meals in our dataset were rare (one bat and one monkey), but their presence is of epidemiological importance, and underscores that even in cityscapes, the presence of nearby wildlife can contribute to host range. Such rare but key events represent critical “ecological bridges” for arboviruses of zoonotic origin [9,21]. *Aedes albopictus* is suspected as a “bridge vector” between urban and sylvatic habitats, which can enable spillover of viruses like yellow fever, dengue, chikungunya, Zika, West Nile, Japanese encephalitis, and Mayaro viruses [42,45,49,50]. Studies in Cameroon and other African regions confirm frequent human feedings and occasional wildlife meals, indicating potential for enzootic–urban transmission, and therefore could facilitate arbovirus amplification and outbreaks [12,51]. While these two non-human blood-meal detections are low to quantify bridge-vector transmission risk, they molecularly notify the characteristic of *Ae. albopictus* feeding across the human–wildlife interface in a peri-urban central African setting. In the context of previous observations supporting the bridge-vector hypothesis for this species, these data add geographically important evidence suggesting that such cross-species feeding events may occur even in urban and peri-urban environments. The detection of nearby wildlife could therefore suggest the need to adopt a One Health approach for arbovirus surveillance in all settings.

### Limitations and Methodological Considerations

Our analysis was based on mosquitoes with visibly blood-filled abdomens, a criterion that underestimates total feeding because partially digested meals or interrupted feedings may go unnoticed. Furthermore, database bias toward human sequences in BLAST searches could inflate anthropophagy estimates. However, this concern is mitigated by the good similarity among sequences (~99%) and their right spotting position on the phylogenetic tree. Of 298 blood-fed *Aedes* females collected, 156 were subjected to COI molecular analysis, and 126 (80.8%) yielded high-confidence host assignments, meeting our quality thresholds (query coverage ≥ 90%, identity ≥ 90%, E-value ≤ 1 × 10^−20^). The 30 failed sequences (19.2%) most likely reflect post-collection blood-meal degradation, PCR inhibition, or sequencing artefacts; limitations common to field-based blood-meal studies. The overall blood-fed rate (5.95%) is consistent with published rates from similar outdoor resting-catch studies in Cameroon and elsewhere in Africa, and does not suggest systematic bias in the recovery of engorged individuals. Nevertheless, this sample size constrains the statistical power available for inferential analysis, and all findings are therefore presented in a descriptive framework with appropriate caution regarding generalisation.

Regarding sampling, our collection methods were not advantageous for *Ae. albopictus* specifically; instead, they targeted common adult resting sites for *Aedes* species in both urban and rural settings, specifically in Cameroon [24]. Importantly, all mosquito collections were done outdoors based on the previous observation in Cameroon. Therefore, future studies should systematically include indoor collections to provide a more complete picture of the relative contributions of both species to indoor and outdoor human biting. Nonetheless, we acknowledge that spatial variability in mosquito abundance and host availability could influence feeding patterns and potentially bias anthropophagy estimates in either direction. Consequently, these findings should be interpreted with caution and not generalised indiscriminately to all assessed contexts. Future studies employing high-throughput sequencing and metabarcoding could improve sensitivity by detecting mixed or partially digested meals [52], providing a more complete picture of mosquito feeding behaviour and pathogen transmission networks.

## 5. Conclusions

Our results show that anthropophagy is the overwhelming pattern in *Ae. albopictus and Ae. aegypti* populations in Cameroon. However, *Ae. albopictus* sporadically feeds on bats and non-human primates, providing preliminary molecular evidence consistent with, though not definitely established, a potential bridge-vector role at urban–wildlife interfaces in Cameroon. These findings are consistent with the global trajectory of *Ae. albopictus* as an adaptable species bridging human and sylvatic ecologies. In the context of accelerating urbanisation and ecological change, routine vector surveillance should prioritise urban and/or rural locations where humans and wildlife intersect, such as city zoos, forest-edge neighbourhoods, and other protected areas embedded within expanding cities, which could serve as interface hotspots for arbovirus spillover and rapid amplification. In addition, genomic surveillance of mosquito blood meals coupled to rigorous ecological monitoring in sentinel sites and protected areas, focusing on mosquito abundance, host communities, presence of wildlife animals, and habitat change, would further enhance a direct, standardised, and scalable measure of host use and support real-time assessment of arboviral emergence or re-emergence risk and prevent future arboviral outbreaks.

## Figures and Tables

**Table 1:** Sites and period of collections.

Locations	Time of Collection	Season	Method Applied	Number of Mosquitoes Collected
Douala (urban)	December 2023	Dry	Prokopack aspirator and BioGents-Sentinel trap	2089
	June 2024	Rainy		
Obala (rural)	June 2023, April 2024	Rainy	Prokopack aspirator and BioGents-Sentinel trap	385
Garoua (urban)	September 2024	Rainy	Prokopack aspirator and BioGents-Sentinel trap	145
Yaoundé (urban)	December 2023	Dry	Prokopack aspirator and BioGents-Sentinel trap	1422
	April, June–July 2023	Rainy		
Yaoundé (Zoo-botanical garden)	November–December 2019	Dry	CDC Back-pack Aspirator	960
	June–August 2020	Rainy		

**Table 2:** List of primers used to amplify the cytochrome oxidase gene.

Primers	Sequences (5′–3′)	Size (bp)	References
COI-forward	ACTTCTGGGTGGCCAAAGAATCAGAA	748	[33]
COI-reverse	TTCTCCAACCACAAAGACATTGGCAC		
Cyt-b-forward	CGAAGCTTGATATGAAAAACCATCGTTG	772	
Cyt-b-reverse	TGTAGTTRTCWGGGTCHCCTA		
COXI-forward	TTCGGCGCATGAGCTGGAGTCC	228	[34]
COX-reverse	TATGCGGGGAAACGCCATATCG		

bp, base pair; COI, vertebrate-specific cytochrome oxidase I; COXI, human-specific cytochrome oxidase I; Cyt-b, mammalian-specific cytochrome b.

**Table 3:** Abundance of blood-fed *Aedes* spp. females in Cameroon.

Location	*Aedes albopictus*		*Aedes aegypti*		Total	
	*n*	nbf (%)	*n*	nbf (%)	*n*	nbf (%)
Douala (urban)	1625	69 (4.24)	464	16 (3.44)	2089	85 (4.06)
Obala (rural)	342	0 (0)	43	0 (0)	385	0 (0)
Garoua (urban)	4	0 (0)	141	0 (0)	145	0 (0)
Yaoundé (urban)	1240	45 (3.62)	182	18 (9.89)	1422	63 (4.43)
Yaoundé (zoo-botanical garden)	960	150 (15.62)	0	0 (0)	960	150 (15.62)
Total	4171	264 (6.32)	830	34 (4.09)	5001	298 (5.95)

*n*, total number of female *Aedes* spp. collected; nbf, number of blood-fed females; %, percentage of blood-fed females.

## Data Availability

Data is contained within the article or Supplementary Material.

## Supplementary Materials

The following supporting information can be downloaded at: https://www.mdpi.com/article/10.3390/tropicalmed11060143/s1, Table S1: Abundance of adult mosquitoes collected in Cameroon. n, total number of female mosquitoes collected; %, percentage calculated over the total number. Table S2: DNA barcoding of *Aedes* blood meals reveals host species composition through COI BLAST identification. Most blood meals were derived from humans (>98%), with rare meals from a monkey (*Papio anubis*) and a fruit bat (Pteropodidae), highlighted in red and blue respectively.
