# Supplementary material for: Anthropophagy and Ecological Bridges: Blood-Meal Patterns of Invasive Aedes albopictus (Skuse, 1894) and Native Aedes aegypti Linnaeus, 1762 and Their Implications for Arbovirus Emergence in Central Africa
Source: Trop Med Infect Dis. 2026 May 25;11(6):143. doi: 10.3390/tropicalmed11060143 (PMC13307752; doi:10.3390/tropicalmed11060143)
Supplement: Supplementary file 1 [file tropicalmed-11-00143-s001.zip › tropicalmed-4262612-supplementary.pdf]

Supplementary Table S1. Abundance of adult mosquitoes collected in Cameroon. n, total number of female mosquitoes collected; %, percentage calculated over the total number.

Locations	Time of collection	Season	*Aedes* spp. n (%)	*Culex* sp. n (%)	*Anopheles* sp. n (%)	*Eretmapodites* sp. n (%)	Total n (%)
Douala (urban)	December 2023	Dry	2,089 (20.78)	1,105 (10.99)	0 (0)	0 (0)	**3,194 (31.77)**
	June 2024	Rainy					
Obala (rural)	June 2023, April 2024	Rainy	385 (3.83)	219 (2.18)	39 (0.39)	17 (0.17)	**660 (6.56)**
Garoua (urban)	September 2024	Rainy	145 (1.4)	2,361 (23.48)	61 (0.61)	0 (0)	**2,567 (25.53)**
Yaoundé (urban)	December 2023	Dry	1,422 (14.14)	613 (6.09)	22 (0.21)	16 (0.15)	**2,073 (20.61)**
	April, June-July 2023	Rainy					
Yaoundé (Zoo-botanical garden)	November-December 2019	Dry	960 (9.55)	600 (5.97)	0 (0)	0 (0)	**1,560 (15.52)**
	June-August 2020	Rainy					
**Total**			**5,001 (49.74)**	**4,898 (48.72)**	**122 (1.21)**	**33 (0.33)**	**10,054 (100)**

Supplementary Table S2. DNA barcoding of *Aedes* blood meals reveals host species composition through COI BLAST identification. Most blood meals were derived from humans (>98%), with rare meals from a monkey (*Papio anubis*) and a fruit bat (Pteropodidae), highlighted in red and blue respectively.

Sequence ID	Accession number of the corresponding reference	Host Common name	Host species name	Query cover	% Identity	E-value	Total score
CmrAlbCOI-F-1	KX456964.1	Human	*Homo sapiens*	100	99.674	0	1122
CmrAlbCOI-F-2	KX456964.1	Human	*Homo sapiens*	100	99.674	0	1122
CmrAlbCOI-F-4	KX456964.1	Human	*Homo sapiens*	100	99.511	0	1118
CmrAlbCOI-F-5	KX456964.1	Human	*Homo sapiens*	100	99.674	0	1122
CmrAlbCOI-F-6	KX456964.1	Human	*Homo sapiens*	100	99.511	0	1118
CmrAlbCOI-F-7	KX456964.1	Human	*Homo sapiens*	100	99.674	0	1122
CmrAlbCOI-F-8	KX456964.1	Human	*Homo sapiens*	100	99.674	0	1122
CmrAlbCOI-F-9	KX456964.1	Human	*Homo sapiens*	100	99.511	0	1116

CmrAlbCOI-F-10	KX456964.1	Human	*Homo sapiens*	100	99.674	0	1122
CmrAlbCOI-F-11	KX456964.1	Human	*Homo sapiens*	100	99.674	0	1122
CmrAlbCOI-F-12	KX456964.1	Human	*Homo sapiens*	100	99.674	0	1122
CmrAlbCOI-F-13	KX456964.1	Human	*Homo sapiens*	100	99.674	0	1122
CmrAlbCOI-F-14	KX456964.1	Human	*Homo sapiens*	100	99.674	0	1122
CmrAlbCOI-F-15	MF058193.1	Human	*Homo sapiens*	100	99.674	0	1122
CmrAlbCOI-F-16	KX456964.1	Human	*Homo sapiens*	100	99.674	0	1122
CmrAlbCOI-F-17	KX456964.1	Human	*Homo sapiens*	100	99.674	0	1122
CmrAlbCOI-F-18	KX456964.1	Human	*Homo sapiens*	100	99.674	0	1122
CmrAlbCOI-F-19	MF055812.1	Human	*Homo sapiens*	100	98.374	0	1090
CmrAlbCOI-F-20	MF058193.1	Human	*Homo sapiens*	100	99.674	0	1122
CmrAlbCOI-F-21	KX456964.1	Human	*Homo sapiens*	100	99.674	0	1122
CmrAlbCOI-F-22	KT698037.1	Human	*Homo sapiens*	100	99.514	0	1122
CmrAlbCOI-F-23	KX456964.1	Human	*Homo sapiens*	100	99.674	0	1122
CmrAlbCOI-F-24	KX456964.1	Human	*Homo sapiens*	100	99.511	0	1118
CmrAlbCOI-F-25	KX456964.1	Human	*Homo sapiens*	100	99.674	0	1122
CmrAlbCOI-F-26	KX456964.1	Human	*Homo sapiens*	100	99.674	0	1122
CmrAlbCOI-F-27	KX456964.1	Human	*Homo sapiens*	100	99.674	0	1122
CmrAlbCOI-F-28	KX456964.1	Human	*Homo sapiens*	100	99.674	0	1122
CmrAlbCOI-F-29	KX456964.1	Human	*Homo sapiens*	100	99.674	0	1122
CmrAlbCOI-F-30	KX456964.1	Human	*Homo sapiens*	100	99.674	0	1122
CmrAlbCOI-F-31	KX456964.1	Human	*Homo sapiens*	100	99.674	0	1122
CmrAlbCOI-F-32	KX456964.1	Human	*Homo sapiens*	100	99.674	0	1122
CmrAlbCOI-F-33	KX456964.1	Human	*Homo sapiens*	100	99.674	0	1122
CmrAlbCOI-F-34	KX456964.1	Human	*Homo sapiens*	100	99.511	0	1118
CmrAlbCOI-F-35	KX456964.1	Human	*Homo sapiens*	100	99.674	0	1122
CmrAlbCOI-F-36	KX456964.1	Human	*Homo sapiens*	100	99.674	0	1122
CmrAlbCOI-F-37	KX456964.1	Human	*Homo sapiens*	100	99.674	0	1122

CmrAlbCOI-F-38	KX456964.1	Human	*Homo sapiens*	100	99.511	0	1118
CmrAlbCOI-F-39	KX456964.1	Human	*Homo sapiens*	100	99.674	0	1122
CmrAlbCOI-F-40	KX456964.1	Human	*Homo sapiens*	100	99.674	0	1122
CmrAlbCOI-F-41	KX456964.1	Human	*Homo sapiens*	100	99.674	0	1122
CmrAlbCOI-F-42	KX456964.1	Human	*Homo sapiens*	100	99.511	0	1118
CmrAlbCOI-F-43	KX456964.1	Human	*Homo sapiens*	100	99.511	0	1116
CmrAlbCOI-F-44	KX456964.1	Human	*Homo sapiens*	100	99.674	0	1122
CmrAlbCOI-F-45	KX456964.1	Human	*Homo sapiens*	100	99.511	0	1116
CmrAlbCOI-F-46	KX456964.1	Human	*Homo sapiens*	100	99.674	0	1122
CmrAlbCOI-F-47	KX456964.1	Human	*Homo sapiens*	100	99.674	0	1122
CmrAlbCOI-F-48	KX456964.1	Human	*Homo sapiens*	100	99.674	0	1122
CmrAlbCOI-F-49	MF055812.1	Human	*Homo sapiens*	100	99.512	0	1116
CmrAlbCOI-F-50	MF055812.1	Human	*Homo sapiens*	100	99.187	0	1109
CmrAlbCOI-F-51	MF055812.1	Human	*Homo sapiens*	100	99.512	0	1116
CmrAlbCOI-F-52	KX456964.1	Human	*Homo sapiens*	100	99.511	0	1118
CmrAlbCOI-F-53	KX456964.1	Human	*Homo sapiens*	100	99.511	0	1118
CmrAlbCOI-F-55	KX456964.1	Human	*Homo sapiens*	100	99.511	0	1118
CmrAlbCOI-F-56	KX456964.1	Human	*Homo sapiens*	100	99.511	0	1118
CmrAlbCOI-F-57	KX456964.1	Human	*Homo sapiens*	100	99.511	0	1116
CmrAlbCOI-F-58	KX456964.1	Human	*Homo sapiens*	100	99.674	0	1122
CmrAlbCOI-F-59	KX456964.1	Human	*Homo sapiens*	100	99.674	0	1122
CmrAlbCOI-F-60	KX456964.1	Human	*Homo sapiens*	100	99.511	0	1118
CmrAlbCOI-F-61	KX456964.1	Human	*Homo sapiens*	100	99.674	0	1122
CmrAlbCOI-F-62	KX456964.1	Human	*Homo sapiens*	100	99.511	0	1118
CmrAlbCOI-F-63	MF055812.1	Human	*Homo sapiens*	100	99.512	0	1116
CmrAlbCOI-F-64	KX456964.1	Human	*Homo sapiens*	100	99.186	0	1110
CmrAlbCOI-F-65	KX456964.1	Human	*Homo sapiens*	100	99.674	0	1122
CmrAlbCOI-F-66	MF055812.1	Human	*Homo sapiens*	100	98.699	0	1098

CmrAlbCOI-F-67	MF055812.1	Human	*Homo sapiens*	100	99.512	0	1116
CmrAlbCOI-F-70	KX456964.1	Human	*Homo sapiens*	100	99.349	0	1114
CmrAlbCOI-F-71	MF055812.1	Human	*Homo sapiens*	100	99.512	0	1116
CmrAlbCOI-F-73	KX456964.1	Human	*Homo sapiens*	100	99.674	0	1122
CmrAlbCOI-F-75	MN849809.1	Human	*Homo sapiens*	100	99.023	0	1098
CmrAlbCOI-F-69	KX456964.1	Human	*Homo sapiens*	100	96.58	0	1048
CmrAlbCOI-F-70	OP682665.1	Human	*Homo sapiens*	100	99.512	0	1116
CmrAlbCOI-F-71	XM_029862027.2	Human	*Homo sapiens*	100	90.667	4.37E-78	305
CmrAlbCOI-F-72	OP682665.1	Human	*Homo sapiens*	100	98.862	0	1101
CmrAlbCOI-F-73	OP682665.1	Human	*Homo sapiens*	100	99.512	0	1116
CmrAlbCOI-F-74	OP682665.1	Human	*Homo sapiens*	100	99.512	0	1116
CmrAlbCOI-F-75	MF055812.1	Human	*Homo sapiens*	100	99.35	0	1110
CmrAlbCOI-F-77	MF055812.1	Human	*Homo sapiens*	100	99.512	0	1116
CmrAlbCOI-F-78	MF055812.1	Human	*Homo sapiens*	100	99.512	0	1116
CmrAlbCOI-F-80	MF055812.1	Human	*Homo sapiens*	100	99.512	0	1116
CmrAlbCOI-F-83	MF055812.1	Human	*Homo sapiens*	100	99.673	0	1116
CmrAlbCOI-F-87	KX456964.1	Human	*Homo sapiens*	100	99.674	0	1122
CmrAlbCOI-F-88	MN849809.1	Human	*Homo sapiens*	100	97.394	0	1070
CmrAlbCOI-F-82	KX456964.1	Human	*Homo sapiens*	100	99.674	0	1122
CmrAlbCOI-F-83	KX456964.1	Human	*Homo sapiens*	100	99.674	0	1122
CmrAlbCOI-F-84	OP682665.1	Human	*Homo sapiens*	100	99.512	0	1116
CmrAlbCOI-F-85	MF055812.1	Human	*Homo sapiens*	100	99.35	0	1112
CmrAlbCOI-F-86	MF057154.1	Human	*Homo sapiens*	100	99.353	0	1118
CmrAlbCOI-F-87	MF058193.1	Human	*Homo sapiens*	100	99.674	0	1122
CmrAlbCOI-F-88	OP682665.1	Human	*Homo sapiens*	100	99.512	0	1116
CmrAlbCOI-F-89	MF055812.1	Human	*Homo sapiens*	100	99.512	0	1116
CmrAlbCOI-F-90	MK248449.1	Human	*Homo sapiens*	100	99.187	0	1109
CmrAlbCOI-F-91	XM_021841368.1	Human	*Homo sapiens*	100	89.944	6.13E-52	219

CmrAlbCOI-F-92	KX456964.1	Human	*Homo sapiens*	100	99.511	0	1118
CmrAlbCOI-F-93	KX456964.1	Human	*Homo sapiens*	100	99.674	0	1122
CmrAlbCOI-F-94	KX456964.1	Human	*Homo sapiens*	100	99.674	0	1122
CmrAlbCOI-F-95	KY409779.1	Human	*Homo sapiens*	100	98.699	0	1098
CmrAlbCOI-F-97	MZ387213.1	Human	*Homo sapiens*	100	99.023	0	1107
CmrAlbCOI-F-98	MF057154.1	Human	*Homo sapiens*	100	99.353	0	1118
CmrAlbCOI-100F	DQ305013.1	Human	*Homo sapiens*	100	99.35	0	1112
CmrAlbCOI-101F	KJ669117.1	Human	*Homo sapiens*	100	99.512	0	1116
CmrAlbCOI-102F	MZ921158.1	Human	*Homo sapiens*	100	99.674	0	1122
CmrAlbCOI-103F	MF057460.1	Human	*Homo sapiens*	100	99.512	0	1116
CmrAlbCOI-104F	DQ305013.1	Human	*Homo sapiens*	100	99.512	0	1116
CmrAegCOI-112F	KJ669117.1	Human	*Homo sapiens*	100	99.187	0	1109
CmrAegCOI-114F	MZ921158.1	Human	*Homo sapiens*	100	99.511	0	1118
CmrAegCOI-115F	MZ921158.1	Human	*Homo sapiens*	100	99.674	0	1122
CmrAegCOI-117F	MZ921158.1	Human	*Homo sapiens*	100	99.674	0	1122
CmrAegCOI-121F	MZ921158.1	Human	*Homo sapiens*	100	98.697	0	1098
CmrAegCOI-124F	MZ387213.1	Human	*Homo sapiens*	100	99.023	0	1107
CmrAegCOI-125F	KJ669117.1	Human	*Homo sapiens*	100	99.512	0	1116
CmrAlboCOI-F-zoo1	MF057449.1	Human	*Homo sapiens*	100	99.35	0	1110
CmrAlboCOI-F-zoo2	MF057449.1	Human	*Homo sapiens*	100	99.35	0	1110
CmrAlboCOI-F-zoo3	KX456964.1	Human	*Homo sapiens*	100	99.674	0	1122
CmrAlboCOI-F-zoo4	KX456964.1	Human	*Homo sapiens*	100	99.674	0	1122
CmrAlboCOI-F-zoo5	KX456964.1	Human	*Homo sapiens*	100	99.674	0	1122
CmrAlboCOI-F-zoo6	KX456964.1	Human	*Homo sapiens*	100	99.674	0	1122
CmrAlboCOI-F-zoo7	KX456964.1	Human	*Homo sapiens*	100	99.674	0	1122
CmrAlboCOI-F-zoo8	KX456964.1	Human	*Homo sapiens*	100	99.674	0	1122
CmrAlboCOI-F-zoo11	OQ538071.1	Monkey	*Papio anubis*	100	98.706	0	1096
CmrAlboCOI-F-zoo16	OP682665.1	Human	*Homo sapiens*	100	99.35	0	1110

CmrAlboCOI-F-zoo17	OP682665.1	Human	*Homo sapiens*	100	99.35	0	1110
CmrAlboCOI-F-zoo18	NC_046913.1	Fruit bat	*Hypsignatus monstrosus*	93	90.97	0	782
CmrAlboCOI-F-zoo35	MK248449.1	Human	*Homo sapiens*	100	99.512	0	1116
CmrAlboCOI-F-zoo37	PV067317.1	Human	*Homo sapiens*	100	98.697	0	1083
CmrAlboCOI-F-zoo50	MN849809.1	Human	*Homo sapiens*	100	99.511	0	1116
CmrAlboCOI-F-zoo67	KX456964.1	Human	*Homo sapiens*	100	99.674	0	1122
